# Policy brief – cancer research in Belgium

**DOI:** 10.1186/s13690-024-01369-3

**Published:** 2024-08-26

**Authors:** Gordana Raicevic Toungouz, Hélène A. Poirel, Gabrielle Schittecatte, Marc Van Den Bulcke, on behalf of the Belgian Cancer Research Alliance, Andries Camille, Beguin Yves, Bekaert Sofie, Berx Geert, Blanpain Cedric, Breckpot Karine, Close Pierre, Constantinescu Stefan, Coosemans An, Coopens Frederik, Custers René, Greve De Jacques, Preter De Katleen, Fuks François, Godderis Lode, Houbracken Isabelle, Kridelka Frederic, Ray Le Veronique, Lembrechts Robrecht, Lobbestael Evy, Lucas Sophie, Machiels Jean-Pascal, Neyns Bart, Noel Agnès, Ost Piet, Peeters Marc, Piccart Martine, Renckens Suzy, Rommel Ward, Rondou Pieter, Sys Gwen, Tejpar Sabine, Bulck Van den Isabelle, Reest Van der Jiska, Gestel Van Dirk, Lint Van Johan, Vanderkerken Karin, Verhelst Xavier, Wozniak Agnieszka, Zimmermann Pascale

**Affiliations:** Department of Epidemiology and Public Health, Cancer Center, Sciensano, Brussels, Belgium

**Keywords:** Research, Innovation, Cancer, Europe’s Beating Cancer Plan, Mission on Cancer

## Abstract

Research is central to achieving Europe’s Beating Cancer Plan, and is the key focus of the European Commission’s Mission on Cancer. To successfully tackle the challenges we face in cancer research, a coordinated effort of the entire Belgian scientific community is needed. It is for this reason the *Belgian Cancer Research Alliance* was proposed. The aim of BeCRA is to bring together various Belgian research institutes and associated care institutions and position them optimally at the EU level, with respect to the many research initiatives launched in the EBCP within the EU4Health program, the Mission on Cancer, Digital Europe programs and other EU projects. Members of BeCRA collaboratively plan the participation in certain cancer research activities to ensure optimal use of investment and sustained excellence of Belgian cancer research. Belgium cancer research has an excellent track record in fundamental, translational research and phase I, II, III clinical trials. However, translating outcomes from research to patients in the Belgian healthcare system has been less successful. Gaps in the collaboration between actors in the research field, have led to fragmentation hampering the development of fundamental and translational research. Moreover, actors from multi-disciplinary background, such as behavioural or psycho-social fields, are not systematically included in cancer research. More efficient coordination between the aforementioned actors is necessary. Academic hospitals and universities should be incentivized to collaborate across regions, as well as to put sufficient focus on research activities with a virtuous spiral ("bed-to bench" and "bench to bed" process), while supporting researchers focusing on patient-driven research. There is an urgent need for Belgium to determine how best to ensure it remains an attractive market so that patients have access to innovative care. This could include streamlining regulatory complexity, while establishing lean and harmonized clinical trial designs, procedures and networks.

## Background

Research is a key element in Europe’s Beating Cancer Plan (EBCP) through its keystone position in the European Commission’s complimentary, research and innovation oriented, Mission on Cancer (MoC) [1]. Following the launch of the EBCP and MoC, Sciensano’s Cancer Centre has taken up responsibilities in research and innovation by supporting actions to bridge the gap between fundamental and translational research, the clinical application of this research, and integrating results into clinical care. The overarching goal being to ensure a direct benefit of research outcomes for patients, their relatives and in some extents, the general population.

In research and innovation at the national level in Belgium, including for cancer research, the government has set an objective of reinforcing its position as a strong “health and biotech valley” [2]. Their aim is to attract a greater share of global biopharmaceutical industry activities, including research, clinical trials, and manufacturing. Achieving this goal will require a joint effort of the government, public services, universities, the entire research community, hospitals and also private sector.

### Main text

#### Methodology

To successfully tackle the challenges faced in cancer research, a coordinated effort of the entire Belgian scientific community is needed. Relatedly, initial discussions of the Belgian-EBCP Mirror Group Thematic Working Group (TWG) on ‘*Research*’, led to the idea of creating an alliance for cancer research in Belgium. This idea that emerged subsequently took shape as the *Belgian Cancer Research Alliance* (BeCRA). The BeCRA Working Group is composed of representatives of the Belgian universities, academic hospitals and their attached research institutes, patient and cancer care organisations, as well as public institutions.

Eight online meetings were organised to discuss the MoC and priorities for BeCRA, cancer research initiatives in Belgium, including related EU calls for funding, and Belgium’s participation based on identified priorities. Consequently, BeCRA action plan was prepared. The gaps, priorities, and recommendations presented in this policy brief reflect the BeCRA Working Group participatory discussions, and serve as an initial overview of the needs and gaps in cancer research in Belgium, which may evolve and be revisited over time with the involvement of new BeCRA members and throughout the implementation of the MoC.

#### Issue overview

Belgium cancer research has an excellent track record in fundamental, translational research and phase I, II, III clinical trials [2].However, translating outcomes from research to patients in the Belgian healthcare system has been less successful. This is in part, a consequence of a lack of structural organization and of adequate funding, which hamper the development of, and access to, innovative cancer patient care. There are also gaps in the collaboration between actors in the research field, leading to fragmentation which impedes the development of fundamental and translational research, and its application in cancer care. Finally, there is a gap in the type of actors and fields involved in cancer research in Belgium. While there is an understanding of the multidisciplinary approaches needed in cancer prevention and diagnosis (e.g., behavioural sciences), treatment (e.g., psycho-social sciences), as well as survivorship, not all these actors are consistently included in research projects or EU calls [3].

#### Projects, gaps & policy recommendations

### Cancer research initiatives

Belgium is currently involved in three European Coordination and Support Actions (CSAs), launched within the Mission on Cancer (MoC). These projects provide opportunities for Belgian research actors to participate in international initiatives, and strengthen the aforementioned gaps in the Belgian research landscape.

“UNCAN.eu” (UNderstand CANcer) refers to a collective European effort that aims to address the barriers in the understanding of cancer’s mechanisms by proposing a structure for a European Federated Cancer Research Data Hubs [4]. “4.UNCAN.eu” was a CSA with a goal to develop a strategic roadmap to launch the UNCAN.eu initiative. By developing a more complete understanding of cancers’ mechanisms from prevention, early diagnosis, and treatment, the initiative will enable the collection of research data, patient health data, and any other relevant data at an unprecedented scale in 6 main areas of research.^1^

A second CSA, “ECHoS” (Establishing Cancer Mission Hubs: Networks and Synergies), envisages the creation of National Cancer Mission Hubs in each Member State and Associated Country [5]. The project’s activities will include raising awareness and coordinating the MoC activities between relevant research and innovation actors at the national, regional and local in the health systems, while also leveraging synergies with EBCP. This will foster policy dialogues between actors on cancer research, and cancer care and control.

The third CSA, “CCI4EU” (Comprehensive Cancer Infrastructures), aims to support Member States/Associated Countries in developing or improving their future or existing CCIs. There will be a particular focus on developing their research innovation and digital-related capacities, and integrating these into cancer care. This project will foster the development of a Belgian network of Comprehensive Cancer Care and Research Infrastructure in order to better integrate research into care for patients, and to guarantee optimal participation in future EU4Health [6] and MoC initiatives.

Despite these initiatives aiming to improve Belgian actors’ participation in EU research opportunities and the development of hubs to foster collaboration between actors, there remains a lack of governance structures linking research actors in Belgium. This hampers the organisation of activities and resources to meet Belgium’s and the MoC cancer research goals efficiently. The consequence of this insufficient collaboration is enhanced by a lack of integration of the activities related to the MoC at national, regional and local levels, including little engagement beyond health system research actors. More efficient coordination between the aforementioned actors is necessary to improve the collaboration of Belgian research groups in responding proactively and effectively to EU calls. Improved response to call will by consequence contribute to improve the international visibility of research activities in Belgium, and impact for patients.

### Belgium research consortium on cancer

In Belgium, BeCRA, a public research consortium supported by researchers, research organizations and public stakeholders in the Belgian research landscape, has been established. Through participation with the activities of the Belgian EBCP Mirror Group, BeCRA’s work will be aligned with the initiatives launched by the EBCP and the Mission on Cancer [7]. Its goals are to guarantee optimal positioning of Belgian cancer researchers at EU level, to ensure long term investment and excellence, to plan future cancer research (and allocate resources), and to support sustained innovation of Belgian cancer care. Although BeCRA members mainly come from private and public research institutes, close collaboration with industry will be necessary to successfully respond to various research calls. The specifics of that cooperation will be discussed within BeCRA.

BeCRA will work to develop a Belgian vision of cancer research aligned with the concepts of Comprehensive Cancer Care Centre (CCC), CCC networks, and Comprehensive Cancer Infrastructures (CCI). The alliance will facilitate the participation of Belgian academic university hospitals and research institutes in EU level initiatives, guaranteeing the optimal positioning of Belgian cancer researchers at EU level. This will contribute to the long term investment to plan cancer research, with resources allocated in a more effective way.

There is an urgent need for Belgium to consider the changes taking place in biomedical research. This includes regulations, determining how best to further strengthen the research ecosystem so that it remains an attractive market for activities such as R&D, clinical trials, the manufacturing of medicines, and the introduction of innovative medicines. BeCRA aims to address some of these needs, by overcoming existing regional barriers and establishing more effective cooperation and coordination between research actors and fostering the integration of innovations into clinical care in Belgium. To enable BeCRA to do so, and ensure its sustainability and impact, several recommendations for decision-makers are proposed in the following section.

### Policy recommend

To address the aforementioned needs, we propose several recommendations for decision-makers, which are detailed below.

- ➢ Stimulate the collaboration and coordination across regions and stakeholders. Incentivize closer collaboration and coordination across regions, among academic hospitals and universities through various mechanisms. For example, earmarking funding in support of collaborative efforts with added value to cancer research, innovation and care leveraging the concept of specialized, multidisciplinary oncological centres.
- ➢ Support a Belgian mapping exercise to highlight the strengths and gaps in the Belgian cancer research ecosystem by identifying areas of expertise.
- ➢ Integrate research into care. Incentivize academic hospitals to put sufficient focus on research activities integrated into clinical care with a virtuous spiral (“bed-to bench” and “bench to bed” process).
- ➢ Reinforce academic, investigator-initiated, and early-phase clinical trials through financial support and strengthening infrastructure.
- ➢ Design programs that build new and validated skills for the future. For example, training programs via e-learning modules, boosting best practices exchange and skills uptake.
- ➢ Streamline regulatory complexity. For example, align implementation modalities of specific regulatory frameworks, harmonized in concrete charters that can be endorsed by the different stakeholders involved, and reinforce the collaboration with HTA and regulatory bodies.
- ➢ Establish lean and harmonized clinical trial designs, procedures and networks across all phases to enable multi-centric clinical trials.
  - In this context, the full implementation of the Clinical Trial Regulation (CTR N° 536/2014) that aims to simplify and harmonize the submission and ethical evaluation process of clinical trials via unique Clinical Trials Information System (CTIS), thus supporting the flow of information between clinical trial sponsors, European Union (EU) Member States, European Economic Area (EEA) countries and the European Commission, is an important step forward.
- ➢ Build capacity and capability of researchers in ‘patient-driven research and how to translate this into practice. For example, integrating unmet patient needs in research agenda settings.
- ➢ Develop training programs for clinical research coordinators. Envision national training courses by all Belgian universities with topics according to their main areas of expertise.
- ➢ Enhance and facilitate access and sharing of data and patient material by leveraging the European Health Data Space**. **For example, harmonize data formatting and implement common interfaces to access data securely, and create platforms to integrate different research-driven data streams, including real-world data. Ensure regular updating and thus the availability of human research materials in existing national biobanks (Belgian Virtual Tumor bank, and BBMRI.be).

## Data Availability

Not applicable.

## Abbreviations

EBCP: Europe’s Beating Cancer Plan
MoC: Mission on Cancer
CSA: Coordination and Support Action
CCI: Comprehensive Cancer Infrastructures
CCC: Comprehensive Cancer Care Centre
HTA: Health Technology Assessment

## Footnotes

1 Cancer prevention, early diagnosis, sensitivity and resistance to therapy, paediatric cancer, cancer and ageing, as well as survivorship.
